# Interleukin‐35 Ameliorates Obesity‐Associated Impairment of Endothelium‐Dependent Vasodilation via the STAT3‐MAPK/ERK‐eNOS Signaling Axis

**DOI:** 10.1002/iid3.70564

**Published:** 2026-10-22

**Authors:** Kai Li, Jie Feng, Ren Gong, Yunman Liu, Yanqing Wu

**Affiliations:** ^1^ Department of Cardiology The Second Affiliated Hospital, Jiangxi Medical College, Nanchang University Nanchang China; ^2^ Jiangxi Key Laboratory of Molecular Medicine, The Second Affiliated Hospital of Nanchang University Nanchang China

**Keywords:** endothelial dysfunction, IL‐35, MAPK/ERK signaling pathway, obesity, STAT3

## Abstract

**Background:**

Obesity impairs endothelial nitric oxide synthase (eNOS)‐nitric oxide (NO) signaling, triggering chronic inflammation, endothelial dysfunction and defective vasodilation, which drives cardiometabolic diseases. Existing metabolic therapies provide only indirect vascular protection and fail to resolve residual inflammatory‐mediated cardiovascular risk. As an anti‐inflammatory interleukin 12 (IL‐12) family cytokine, interleukin 35 (IL‐35) is suppressed in obesity and exerts vascular protective effects under inflammatory conditions, yet its function and underlying mechanisms in obesity‑related endothelial vasodilator dysfunction remain undefined.

**Materials and Methods:**

An obese mouse model was established by 8‐week high‐fat diet (HFD) feeding, followed by 4‐week intraperitoneal administration of recombinant mouse IL‐35. Aortic‐ring assays evaluated vasodilatory function. Palmitic acid (PA)‐treated human umbilical vein endothelial cells (HUVECs), recapitulate lipotoxic endothelial injury, were treated with recombinant human IL‐35. Western blotting, immunofluorescence, 4‐amino‐5‐methylamino‐2′,7′‐difluorofluorescein diacetate (DAF‐FM DA) detection and immunoprecipitation‐mass spectrometry (IP‐MS) were applied to detect IL‐35‐subunit (Epstein‐Barr virus‐induced gene 3 [EBI3] and interleukin‐12 subunit alpha [IL‐12A]) levels, eNOS Ser1177 phosphorylation, phosphorylation of mitogen‐activated protein kinase/extracellular signal‐regulated kinase (MAPK/ERK) signaling molecules (cellular rapidly accelerated fibrosarcoma [cRAF], mitogen‐activated protein kinase kinase [MEK], and ERK) and signal transducer and activator of transcription 3 (STAT3) activation, NO production, and eNOS‐binding partners. Rescue assays were performed using Raf or ERK specific inhibitors and STAT3 small interfering RNA (siRNA).

**Results:**

HFD mice displayed reduced aortic endothelial EBI3 expression, impaired endothelium‐dependent vasorelaxation, decreased eNOS Ser1177 phosphorylation and lowered NO production; all these phenotypes were rescued by exogenous IL‐35. IP‐MS and immunoblotting demonstrated that IL‐35 strengthened eNOS‐ERK1 interaction and restored PA‐repressed phosphorylation of cRAF, MEK, ERK and eNOS Ser1177, which were abrogated by Raf or ERK blockade. Furthermore, IL‐35 recovered STAT3 phosphorylation suppressed by PA, while STAT3 knockdown blunted IL‐35‐driven MAPK/ERK activation, eNOS phosphorylation and subsequent NO production.

**Conclusion:**

IL‐35 alleviates obesity‐induced impairment of endothelium‐dependent vasodilation via the STAT3‐MAPK/ERK‐eNOS axis, which elevates eNOS Ser1177 phosphorylation and NO synthesis. This work supports IL‐35 as a prospective therapeutic candidate for obesity‐associated vascular complications.

## 1 Introduction

Obesity poses a major global public health burden. In 2022, an estimated 504 million women and 374 million men worldwide lived with obesity [1], which markedly elevates susceptibility to type 2 diabetes, hypertension, atherosclerosis, non‐alcoholic fatty liver disease, sleep apnea, osteoporosis and multiple cancers. Obesity‐triggered endothelial dysfunction is a central pathological driver of these disorders [2].

Endothelial cells form the luminal vascular monolayer and sustain vascular homeostasis mainly through eNOS, the rate‐imiting enzyme for endothelial NO production. The eNOS‐NO axis confers broad vascular protection: NO mediates endothelium‐dependent vasodilation, inhibits platelet aggregation and adhesion, restrains vascular smooth‐muscle (VSMC) overgrowth, and exerts anti‐inflammatory and anti‐oxidant actions to maintain endothelial integrity. eNOS activity is tightly controlled by post‐translational modifications; phosphorylation at Ser1177 is a critical activating modification that substantially increases enzymatic activity and enables rapid endothelial responses to physiological cues [3, 4]. Under pathological stress such as hyperlipidaemia, hyperglycemia and inflammation, eNOS activity and NO bioavailability decline. This disturbance promotes vasoconstriction, inflammation, oxidative stress, aberrant proliferation, coagulation dysregulation and adhesion‐molecule upregulation [5, 6, 7, 8, 9], resulting in endothelial dysfunction that accelerates cardiometabolic disease progression.

Obesity‐associated chronic low‐grade inflammation drives endothelial injury and cardiovascular complications independent of body weight and metabolic indices. Current anti‐obesity and lipid‐lowering agents (e.g., liraglutide, orlistat, statins) confer partial endothelial benefits secondary to improved weight, glucose and lipid profiles [10, 11, 12, 13, 14]. However, their vascular protection is largely indirect and metabolism‐dependent. Many patients still exhibit chronic inflammation and endothelial dysfunction despite optimal metabolic control, leaving residual cardiovascular risk. Therefore, therapies directly targeting the vascular inflammatory microenvironment and eNOS function remain an unmet clinical need.

IL‐35 is an anti‐inflammatory IL‐12 family cytokine predominantly secreted by regulatory T cells (Tregs). Circulating IL‐35 is reduced in obesity, likely owing to Treg functional exhaustion [15, 16]. Pre‐clinical studies demonstrate that IL‐35 protects against atherosclerosis, pulmonary hypertension, ischemic vascular injury and autoimmune inflammation by dampening inflammatory and endothelial activation, regulating angiogenesis and inhibiting endothelial‐to‐mesenchymal transition [17, 18, 19, 20]. Nevertheless, it remains unclear whether IL‐35 can reverse obesity‐induced impairment of endothelium‐dependent vasodilation and which intracellular signaling cascades mediate such vascular protection.

Herein, we explored the protective effects of IL‐35 against obesity‐related endothelial vasodilator dysfunction and characterized the STAT3‐MAPK/ERK‐eNOS signaling axis mediating these effects, aiming to fill the knowledge gap for IL‐35 as a candidate therapeutic target for metabolic endothelial injury.

## 2 Materials and Methods

### 2.1 Materials

Thirty male C57BL/6 J mice were purchased from Vital River Laboratory Animal Technology (China). HUVECs were purchased from Procell Life Science & Technology (China). Reagents included PA (AAPR156‐EA1, Pythonbiotech, China), recombinant mouse IL‐35 (PRP1307‐C, Abbkine, China), recombinant human IL‐35 (8608‐IL, R&D Systems, USA), IL‐35 enzyme‐linked immunosorbent assay (ELISA) kits (MM‐0904M1, Meimian Industry, China), and DAF‐FM DA NO assay kits (S0020S, Beyotime, China). Physiological saline solution (PSS) and high‐potassium PSS (KPSS) were prepared in‐house; full formulations are provided in the Supporting materials.

Primary antibodies for immunofluorescence and Western blotting: EBI3 (12371‐1‐AP), IL‐12A (85754‐2‐RR), platelet endothelial cell adhesion molecule‐1 (PECAM‐1/CD31) (11265‐1‐AP), eNOS (27120‐1‐AP), and β‐tubulin (66240‐1‐Ig) from Proteintech (USA); p‐eNOS (Ser1177, #9571), p‐cRAF (#9427), p‐MEK1/2 (#9154), and p‐STAT3 (#9145) from Cell Signaling Technology (USA); cRAF (A19638), MEK1/2 (A4868), p‐ERK1/2 (AP0974), ERK1/2 (A4782), and STAT3 (A1192) from ABclonal Technology (China). HRP‐conjugated secondary antibodies (goat anti‐mouse IgG, SA00001‐1; goat anti‐rabbit IgG, SA00001‐2) were sourced from Proteintech (USA).

### 2.2 Cell Treatment

HUVECs (passages 4–6) were cultured in endothelial cell medium (Sciencell, #1001, USA) at 37°C with 5% CO_2_. Lipotoxic injury was induced by palmitic acid (PA). For dose‐finding, cells seeded in 96‐well plates were treated with PA (0, 12.5, 25, 50, 100, 200 μM) for 24 h. Cell viability was assessed by cell counting kit 8 (CCK‐8) assay (Solarbio, CA1210, China); absorbance was read on a SuPerMax 3500 microplate reader (Flash, China). Based on CCK‐8 results (Figure S2), 100 μM PA was selected for subsequent experiments. Two doses of recombinant human IL‐35 (50 ng/mL and 100 ng/mL) showed comparable protective effects against PA‐induced injury. Consistent with our prior work [20, 21], 100 ng/mL IL‐35 was adopted for mechanistic rescue assays to guarantee adequate dynamic range for phosphorylation detection. HUVECs were assigned to the following groups: control, PA (100 μM), PA + IL‐35 (50 ng/mL), PA + IL‐35 (100 ng/mL), PA + IL‐35 + sorafenib (5 μmol/L, Raf inhibitor, MCE, USA), PA + IL‐35 + SCH772984 (0.5 μmol/L, ERK inhibitor, MCE, USA), PA + IL‐35 + si‐NC (50 nM), and PA + IL‐35 + si‐STAT3 (50 nM), with a total treatment duration of 24 h. PA, IL‐35 and inhibitors were added simultaneously for the control, PA, PA + IL‐35, PA + IL‐35+sorafenib and PA + IL‐35 + SCH772984 groups. For si‐NC and si‐STAT3 groups, cells were transfected for 24 h before co‐incubation with PA and IL‐35 for another 24 h.

### 2.3 Animal Treatment

Eight‐week‐old male C57BL/6 J mice (*n* = 30) were housed under specific‐pathogen‐free (SPF) conditions at the Biomedical Testing Center of Nanchang University (23 ± 2°C, 53 ± 2% humidity) with free access to food and water. After 1‐week acclimatization, mice were randomly divided into three groups (*n* = 10 per group): control, high‐fat diet (HFD), and HFD + IL‐35. Group size was determined from prior similar studies without pre‐study power calculation. The control group received standard chow, whereas the HFD and HFD + IL‐35 groups were fed a high‐fat diet containing 60% fat for 8 weeks to induce obesity. Body weight was monitored weekly; fasting blood glucose (FBG) and random blood glucose (RBG) were detected at week 8. Compared with controls, HFD‐fed mice showed > 1.2‐fold higher body weight and elevated blood glucose (*p* < 0.05) (Figure S1), confirming successful obesity model establishment.

After model validation, mice continued their respective diets for another 4 weeks. During this intervention period, the HFD + IL‐35 group received intraperitoneal injections of recombinant mouse IL‐35 (0.5 μg/g body weight diluted in phosphate‐buffered saline [PBS]) weekly; control and HFD groups were administered an equal volume of PBS [6, 22]. Twenty‐4 h following the final intraperitoneal injection, mice underwent thoracotomy under isoflurane anesthesia for tissue and serum collection. No pre‐defined inclusion/exclusion criteria were applied, and all mice completing the protocol were included in final analyses. All animal procedures were approved by the Nanchang University Laboratory Animal Ethics Committee and performed following the NIH Guidelines for the Care and Use of Laboratory Animals (Publication No. 85‐23, revised 1985) and the ARRIVE guidelines.

### 2.4 Immunofluorescence Colocalization

Frozen aortic Section (6 µM) were subjected to immunofluorescence staining. Sections were blocked with 5% bovine serum albumin (BSA) and incubated overnight at 4°C with primary antibodies against CD31 (1:100), EBI3 (1:100), and IL‐12A (1:100). Subsequently, sections were incubated with Alexa Fluor‐conjugated secondary antibodies (1:200) for 1 h at room temperature. Nuclei were counterstained with 4′,6‐diamidino‐2‐phenylindole (DAPI). Image acquisition was performed using a confocal microscope. At least five random, non‐overlapping high‐power fields within the vascular intima were analyzed for each aortic section. The fluorescence intensity of EBI3 and IL‐12A colocalized with CD31 was quantified using ImageJ, and the mean intensity calculated from all fields per mouse served as a single biological replicate.

### 2.5 Collection of Mouse Aortic Tissue and Serum

Twenty‐4 h after the final intraperitoneal injection, mice underwent thoracotomy under isoflurane anesthesia. One‐milliliter blood samples were collected from the cardiac apex into heparin‐free tubes. Serum was obtained by centrifugation at 3000 g for 15 min at 4°C, aliquoted, and stored at −80°C for biochemical and ELISA assays. Mice were euthanized by gradual inhalation of 100% CO_2_, with ventilation sustained for over 1 min after respiratory arrest. Aortas were harvested within 5 min upon confirmation of cardiac arrest and pupil dilation. A 2‐mm thoracic aortic segment was dissected, with perivascular fat and connective tissue carefully removed under a microscope, and incubated in oxygenated PSS for vascular‐ring assays. The aortic arch was fixed in 4% paraformaldehyde for paraffin sectioning, and remaining aortic tissue was flash‐frozen and kept at −80°C for protein extraction.

### 2.6 Vascular Ring Experiment

Vasodilatory function of aortic rings was assessed using a multi‐channel microvascular tension system (DMT‐620M; Danish Myo Technology, Denmark). Isolated mouse aortic rings were incubated in oxygenated PSS at 37°C until baseline tension stabilized. Vascular viability was verified by pre‐contraction with KPSS followed by washing with PSS. Vessels were pre‐contracted to a stable plateau with 1 μM norepinephrine (NE; Solarbio, IN0530, China). Cumulative acetylcholine (ACh; Solarbio, G8320, China; 10^‐9^−10^‐4^ M) was applied to determine endothelium‐dependent relaxation. After washing and repeated NE‐induced pre‐contraction, sodium nitroprusside (SNP; Solarbio, 9560, China) was added cumulatively over the same concentration range for endothelium‐independent relaxation assessment. Tension responses were recorded for each concentration to generate concentration‐response curves.

### 2.7 Blood Biochemistry and ELISA Assays

Serum triglyceride (TG), total cholesterol (TC), high‐density lipoprotein cholesterol (HDL‐C), and low‐density lipoprotein cholesterol (LDL‐C) were measured using a BIOBASE BK‐280 automated biochemical analyzer following the manufacturer's protocol. Serum IL‐35 concentrations were quantified by ELISA against kit‐supplied standard curves.

### 2.8 Transfection and Quantitative Real‐Time PCR (qRT‐PCR)

Cells were seeded in 6‐ or 24‐well plates and transfected at ~50% confluence with 50 nmol/L STAT3‐targeting siRNA (si‐STAT3) or negative control siRNA (si‐NC; Riobio, China) using CALNP RNAi reagent (DN001‐05; d‐Nano Therapeutics, China) per the manufacturer's protocol. Three independent si‐STAT3 constructs were screened and validated by Western blotting and qRT‐PCR (Figure S4); si‐STAT3‐3 was selected for subsequent experiments based on its highest knockdown efficiency and most stable GAPDH Ct values. Twenty‐4 h post‐transfection, total RNA was extracted with an EZBioscience RNA kit (USA) and reverse‐transcribed using Prime Script RT Kit (Takara, Japan). qRT‐PCR was performed on an Eastwin eQ. 9600 system (China) with Takara SYBR Green Premix Ex Taq. GAPDH served as the internal reference gene, with stable expression confirmed by an overall Ct coefficient of variation of 2.10% and a maximum intergroup mean Ct difference of 0.78 cycles (Table S1). Gene expression was quantified via the 2^−^ΔΔCt method. In parallel, transfected cells were co‐treated with PA and IL‐35 24 h post‐transfection, and subjected to further analyses after another 24 h. siRNA and primer sequences, synthesized by Riobio (China), are provided in Supporting materials.

### 2.9 Cellular Nitric Oxide Assay

Cells were seeded in 24‐well plates and treated upon reaching 70% confluence. The experimental groups included control, PA (100 μM), PA + IL‐35 (50 ng/mL), PA + IL‐35 (100 ng/mL), PA + IL‐35 + Raf inhibitor (5 μmol/L), PA + IL‐35 + ERK inhibitor (0.5 μmol/L), PA + IL‐35 + si‐NC (50 nmol/L), and PA + IL‐35 + si‐STAT3 (50 nmol/L). After 24 h of treatment, culture medium was aspirated and wells were rinsed once with PBS. Protected from light, DAF‐FM DA working solution (5 μM, diluted from 5 mM stock in detection buffer) was added at 200 μL per well and incubated at 37°C for 20 min in the dark. The solution was then removed, wells were washed three times with PBS, and 200 μL PBS was added for immediate visualization and imaging under a fluorescence microscope.

### 2.10 Western Blot

Total protein was extracted from mouse aortic tissue and HUVECs and quantified using the BCA Protein Quantification Kit (Solarbio, China). For each sample, 40 μg of protein was separated by 8% sodium dodecyl sulfate‐polyacrylamide gel electrophoresis (SDS‐PAGE) and transferred to polyvinylidene difluoride (PVDF) membranes (Millipore, USA). Membranes were blocked with 5% BSA for 1 h, and then incubated overnight at 4°C with primary antibodies against EBI3, IL‐12A, eNOS, β‐tubulin, p‐eNOS (Ser1177), p‐cRAF, p‐MEK1/2, p‐ERK1/2, p‐STAT3, cRAF, MEK1/2, ERK1/2, and STAT3 (all at 1:1000 dilution). Following incubation with HRP‐conjugated goat anti‐rabbit IgG and goat anti‐mouse IgG secondary antibodies (both 1:5000), protein bands were analyzed using Image Lab 6.0 software (BioRad, USA). For phosphorylated protein detection, membranes were first probed for phosphorylated targets, and then stripped to remove primary antibodies and re‐probed for corresponding total proteins using the same protocol.

### 2.11 Statistical Analysis

All experiments were conducted with independent biological replicates: cell experiments were repeated at least three times using different cell batches, and animal experiments were repeated with distinct animals. Statistical analyses were performed using SPSS version 26.0 (IBM, USA). Quantitative data are expressed as mean ± standard deviation (SD). One‐way analysis of variance (one‐way ANOVA) was used for multi‐group comparisons, and t‐tests were used for two‐group comparisons. Concentration‐response curves were analyzed by two‐way ANOVA followed by Fisher's Least Significant Difference (LSD) post‐hoc tests. A *p* < 0.05 was considered statistically significant. Investigators performing vascular ring assays, Western blotting, immunofluorescence imaging, and data quantification were blinded to group allocation.

## 3 Results

### 3.1 Development of a Diet‐Induced Obesity Model and the Impact of IL‐35 on Serum Lipid Profiles

We monitored body 8 weekly, measured fasting and random blood glucose at week 8, and assessed serum lipid profiles (TG, TC, LDL‐C, HDL‐C) at week 12 upon euthanasia. By Week 8, both HFD‐fed groups exhibited a more than 1.2‐fold increase in body weight and markedly elevated blood glucose levels compared with controls, confirming successful induction of diet‐induced obesity [23] (Figure S1A, B). At week 12, the HFD group showed significantly elevated levels of all measured lipids compared with the NC group (Figure 1A). IL‐35 treatment significantly reduced serum TG levels but did not alter TC, LDL‐C, or HDL‐C levels (Figure 1A). These findings indicate that IL‐35 selectively reduces circulating triglycerides without affecting cholesterol metabolism.

**Figure 1:**
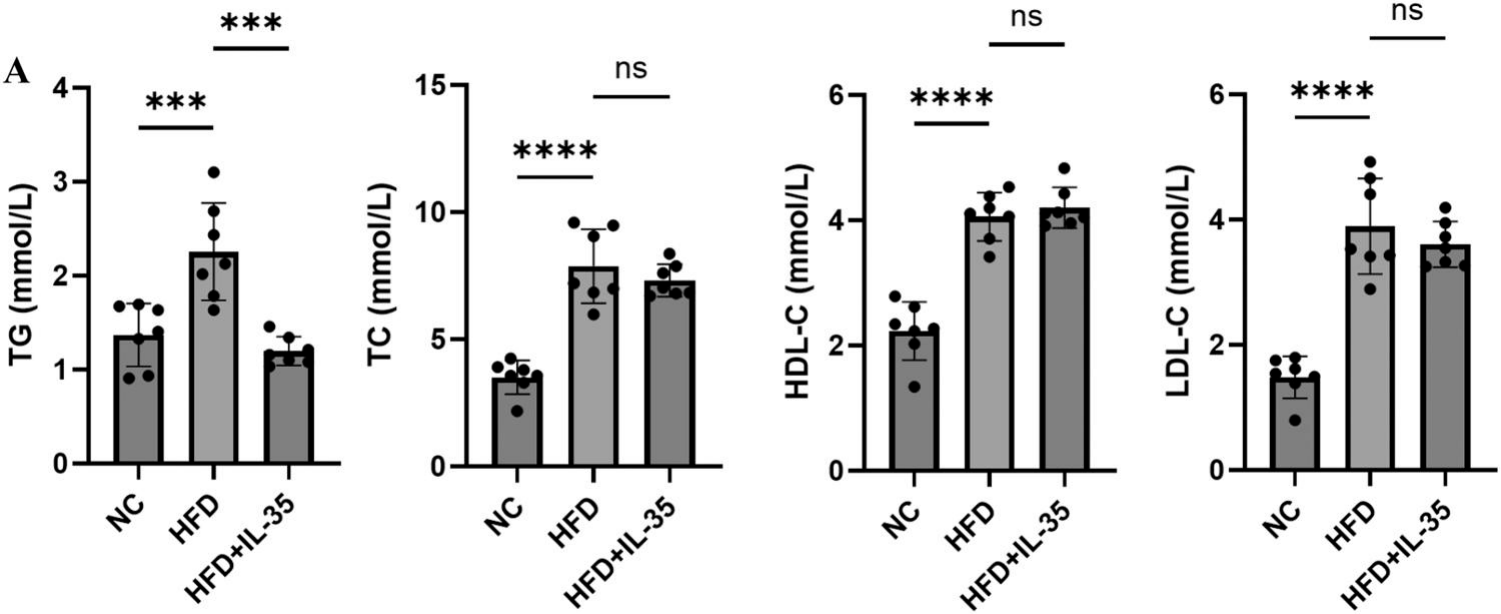
The Impact of IL‐35 on Serum Lipid Profiles. (A) Serum lipid profiles including TG, TC, LDL‐C, and HDL‐C, *n* = 7. Data are mean ± SD. ns, not significant; ***p < 0.001, ****p < 0.0001.

### 3.2 IL‐35 Subunit EBI3 Is Downregulated in Both Obese Mouse Aortas and PA‐Treated Huvecs, and Restored by Exogenous IL‐35

Immunofluorescence colocalization was performed to evaluate EBI3 and IL‐12A expression in aortic endothelial cells (marked by CD31) across the NC, HFD, and HFD + IL‐35 groups. CD31 expression was downregulated in HFD aortic endothelium and restored by IL‐35 treatment, consistent with our prior findings in an LPS‐induced endothelial injury model [20]. Corresponding quantitative data is not presented to avoid redundancy.

Regarding IL‐35 subunits, EBI3 fluorescence intensity in aortic endothelial cells was notably reduced in the HFD group versus controls, and elevated by IL‐35 supplementation (Figure 2A,B). Western blotting confirmed these EBI3 protein expression trends (Figure 2F,H). In contrast, IL‐12A expression showed no significant differences across the three groups by either assay (Figure 2C,D,F,H). ELISA further revealed significantly lower serum IL‐35 levels in the HFD group (Figure 2E). PA, a predominant saturated fatty acid accounting for ~38% of circulating saturated fatty acids in obesity [24], induces lipotoxic endothelial injury that largely recapitulates HFD‐induced effects [25, 26, 27]. In PA‐treated HUVECs, EBI3 protein expression was significantly decreased compared with controls, and substantially upregulated by 100 ng/mL IL‐35 treatment (Figure 2G,I). IL‐12A expression remained unchanged across groups, consistent with in vivo findings (Figure 2G,I).

**Figure 2:**
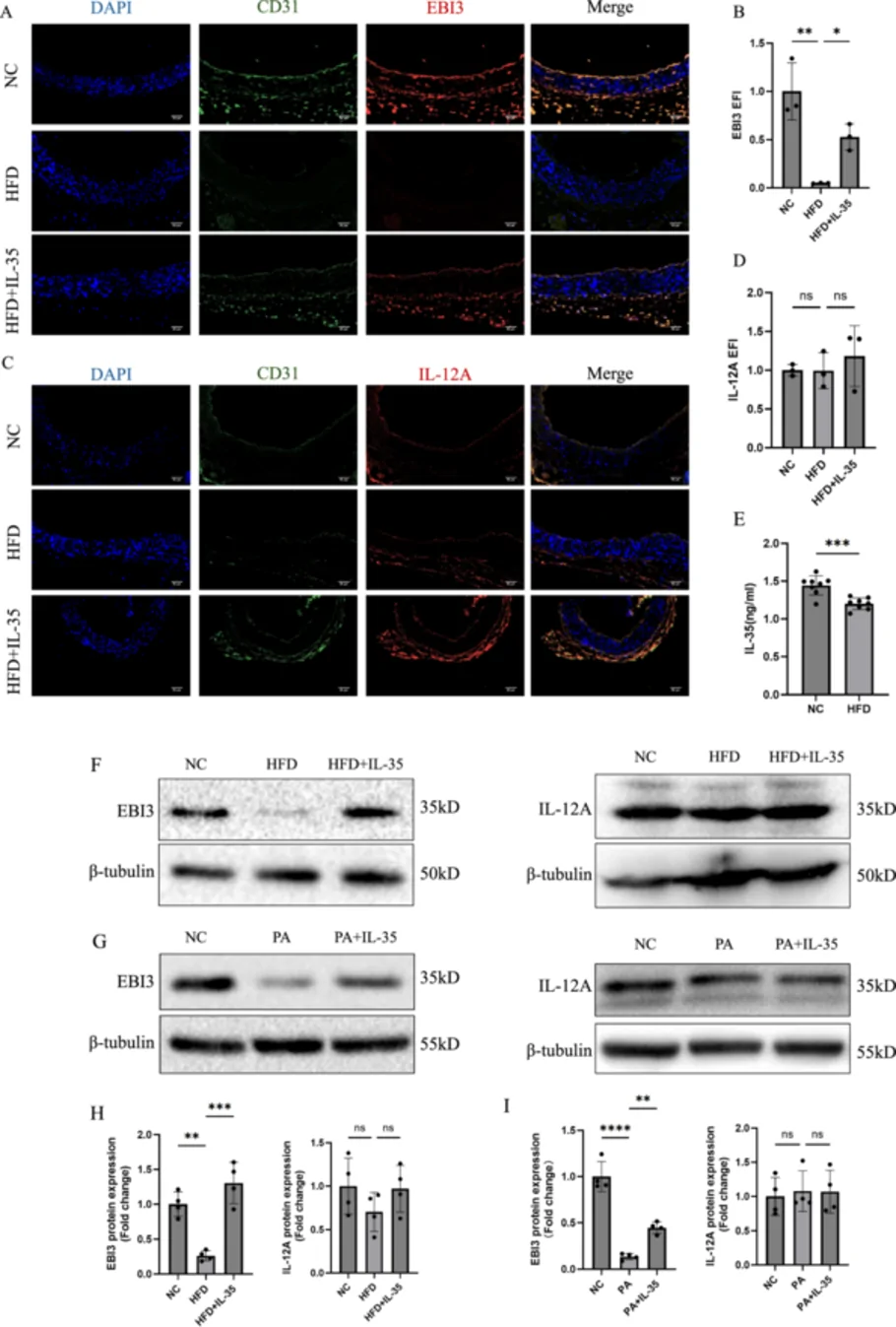
IL‐35 Subunit EBI3 is Downregulated in both Obese Mouse Aortas and PA‐Treated HUVECs, and Restored by Exogenous IL‐35. (A, B) Immunofluorescence of EBI3 (red) with CD31 (green) and DAPI (blue) in mouse aortas, with semi‐quantification of fluorescence intensity, *n* = 3 (scale bar = 50 µM). (C, D) Immunofluorescence of IL‐12A (red) with CD31 (green) and DAPI (blue) in mouse aortas, with semi‐quantification of fluorescence intensity, *n* = 3 (scale bar = 50 µM). (E) Serum IL‐35 concentrations, *n* = 8. (F, H) Representative Western blot bands of EBI3 and IL‐12A in aortic tissues (F) and densitometric analysis (H, *n* = 4). (G, I) Representative Western blot bands of EBI3 and IL‐12A in HUVECs (G) and densitometric analysis (I, *n* = 4). Data are mean ± SD. ns, not significant; *p < 0.05, **p < 0.01, ***p < 0.001, ****p < 0.0001.

Collectively, obesity is linked to reduced systemic IL‐35 levels and suppressed endothelial EBI3 expression. Exogenous IL‐35 selectively upregulates EBI3 in both aortic endothelial cells and HUVECs, with no significant effect on IL‐12A expression.

### 3.3 IL‐35 Restores eNOS Ser1177 Phosphorylation and Nitric Oxide Production to Enhance Endothelium‐Dependent Relaxation in Obesity

To investigate whether IL‐35 improves vasodilation in obese mice, we performed in vitro aortic ring assays [28]. ACh was used to assess endothelium‐dependent vasodilation, and SNP for endothelium‐independent vasodilation. Compared with controls, HFD mice exhibited significantly reduced endothelium‐dependent vasodilation in response to ACh, with a decrease in maximum relaxation (Figure 3A). IL‐35 treatment markedly improved this vasodilatory function (Figure 3A). Endothelium‐independent vasodilation was also reduced in HFD mice, but IL‐35 showed a non‐significant trend of improvement (Figure 3B). These data indicate that exogenous IL‐35 predominantly ameliorates obesity‐impaired endothelium‐dependent vasodilation.

**Figure 3:**
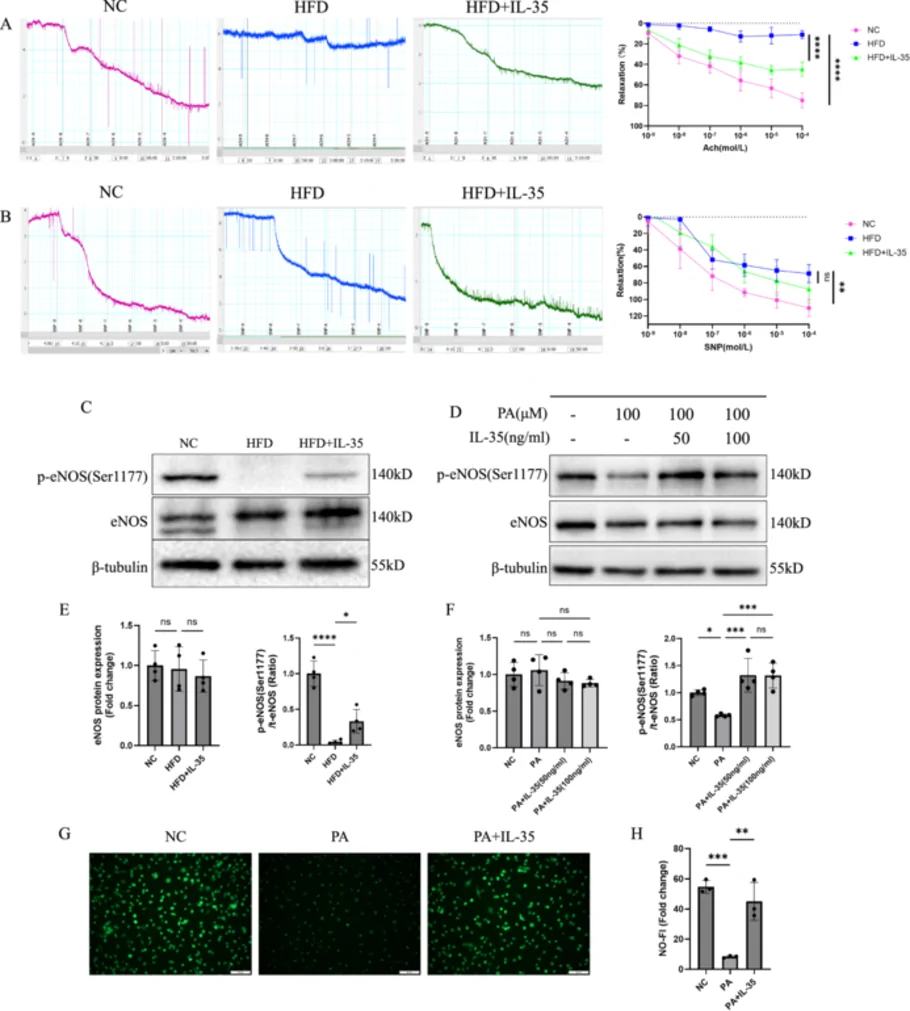
IL‐35 Restores eNOS Ser1177 phosphorylation and nitric oxide production to enhance endothelium‐dependent relaxation in obesity. (A) Concentration‐response curves of endothelium‐dependent relaxation to ACh and comparison of maximal relaxation (Emax) in mouse aortic rings, *n* = 4. (B) Concentration‐response curves of endothelium‐independent relaxation to SNP and comparison of Emax, *n* = 4. (C, D) Western blot bands of p‐eNOS (Ser1177) and total eNOS in mouse aortic tissues (C) and HUVECs (D). (E, F) Densitometric analysis of total eNOS and p‐eNOS (Ser1177)/total eNOS in aortic tissues (E, *n* = 4) and HUVECs (F, *n* = 4). (G) NO fluorescence intensity in HUVECs, scale bar = 50 μM. (H) Semi‐quantification of NO fluorescence intensity in HUVECs, *n* = 3. Data are mean ± SD. ns, not significant; *p < 0.05, **p < 0.01, ***p < 0.001, ****p < 0.0001.

Endothelial cells maintain vascular homeostasis via NO production, which mediates vasodilation, inhibits platelet aggregation, and suppresses inflammation. eNOS is the rate‐limiting enzyme for endothelial NO synthesis, and its activity is tightly regulated by post‐translational modifications [29, 30]. Phosphorylation at Ser1177 is a key activating modification that increases eNOS activity by several‐ to ten‐fold, representing a primary mechanism for endothelial responses to physiological stimuli [3, 4].

Western blotting of mouse aortic tissue showed that eNOS Ser1177 phosphorylation was significantly reduced in HFD mice compared with controls, and was restored by IL‐35 treatment. Total eNOS protein levels showed no significant differences across groups (Figure 3C,E). Consistently, in PA‐treated HUVECs, eNOS Ser1177 phosphorylation was significantly decreased and restored by IL‐35 (50 and 100 ng/mL), with no significant differences in total eNOS levels (Figure 3D,F). NO fluorescence assays further confirmed that the reduction in NO production induced by PA was significantly reversed by IL‐35 (Figure 3G,H).

Collectively, these results demonstrate that IL‐35 mitigates lipotoxic endothelial dysfunction by enhancing eNOS Ser1177 phosphorylation and increasing NO production.

### 3.4 IL‐35 Induces the MAPK/ERK Signaling Pathway, Thereby Enhancing eNOS Ser1177 Phosphorylation and NO Production in Endothelial Cells

eNOS Ser1177 phosphorylation is modulated by multiple upstream signaling pathways: shear stress and VEGF promote its phosphorylation via the PI3K/Akt pathway [31, 32], ischemia and physical exercise activate AMPK [33], prostacyclin and bradykinin stimulate PKA [34], and activation of the MAPK/ERK pathway also ehances Ser1177 phosphorylation [35, 36].

To clarify the upstream regulatory mechanism of IL‐35‐induced eNOS Ser1177 phosphorylation, we characterized the eNOS interactome in PA‐treated HUVECs with or without IL‐35 via IP‐MS. Volcano plot analysis revealed significant changes in the abundance of several eNOS‐binding proteins in the IL‐35‐treated group. Notably, the interaction with ERK1 (MAPK3) showed significant increase (fold change > 1.5, *p* < 0.05, Figure 4A). STRING analysis of the top 20 differentially bound proteins confirmed that only ERK1 directly interacts with eNOS among these candidates (Figure 4B). These findings indicate that IL‐35 enhances eNOS Ser1177 phosphorylation by activating the MAPK/ERK signaling pathway. Of note, this STRING network integrates eNOS‐interacting proteins identified by our IP‐MS assay together with upstream pathway regulators. STAT3 was not detected as a direct eNOS‐binding partner in the IP‐MS analysis, and its regulatory function is validated in the subsequent functional experiments.

**Figure 4:**
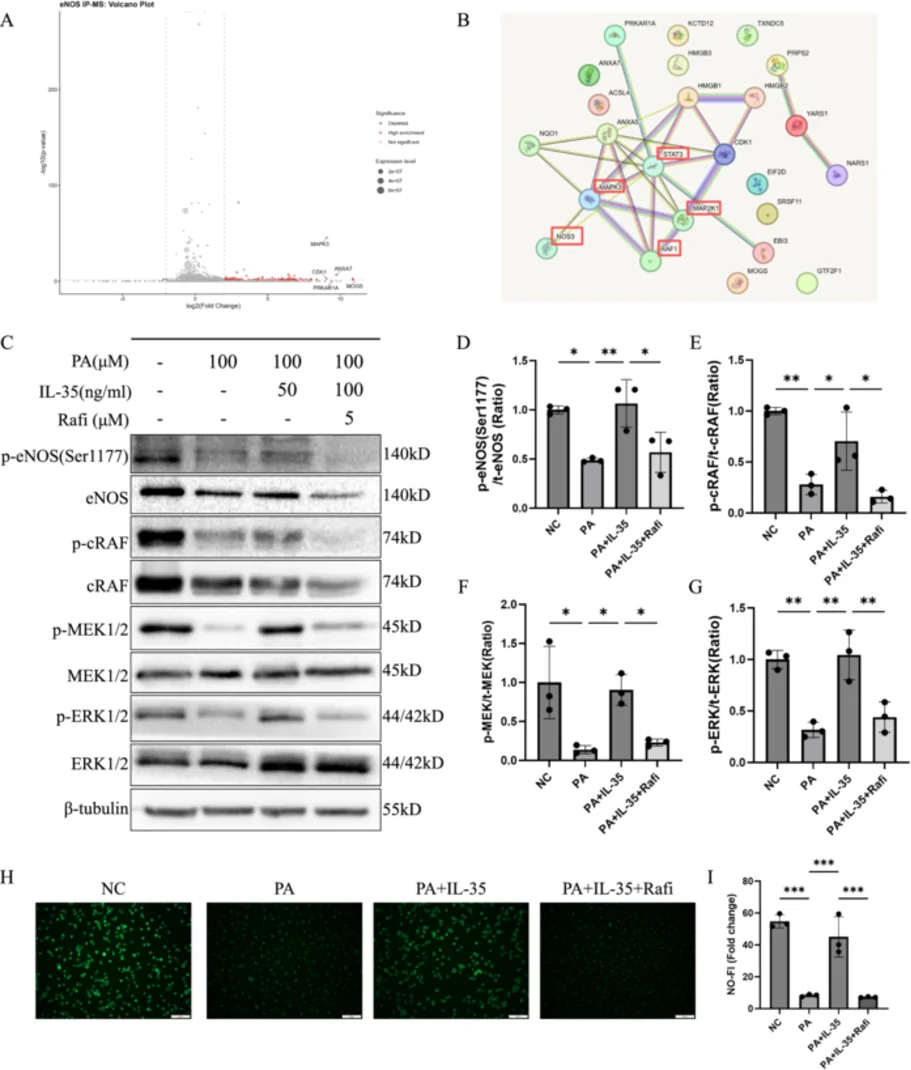
IL‐35 induces the MAPK/ERK signaling pathway, thereby enhancing eNOS Ser1177 phosphorylation and NO production in endothelial cells. (A) Volcano plot of eNOS‐interacting proteins in HUVECs (PA vs. PA + IL‐35) by IP‐mass spectrometry. Red dots: upregulated in PA + IL‐35 group (fold change > 1.5, *p* < 0.05); MAPK3 (ERK1) is highlighted. (B) STRING‐based PPI network of top 20 binding proteins. ERK1 is the only direct interactor with eNOS. (C) Western blot bands of p‐cRAF, p‐MEK, p‐ERK, p‐eNOS (Ser1177), and total proteins in HUVECs. (D–G) Densitometric analysis of p‐cRAF, p‐MEK, p‐ERK, and p‐eNOS (Ser1177) normalized to total proteins, *n* = 3. (H, I) NO fluorescence intensity in HUVECs (H, scale bar = 50 μM) with semi‐quantification (I, *n* = 3). Data are mean ± SD. *p < 0.05, **p < 0.01, ***p < 0.001.

We then performed rescue experiments with the Raf inhibitor sorafenib (5 μM) [37, 38], HUVECs were categorized into four groups: NC, PA, PA + IL‐35, and PA + IL‐35+Rafi. Western blotting showed that PA treatment significantly reduced phosphorylation of cRAF, MEK, ERK, and eNOS compared with controls; IL‐35 treatment markedly elevated the phosphorylation levels of these proteins, which were effectively reversed by concurrent Raf inhibition (Figure 4C–G). NO fluorescence imaging further confirmed that Raf inhibition attenuated IL‐35‐induced NO release from endothelial cells (Figure 4H,I), reinforcing the pivotal role of the MAPK/ERK pathway in IL‐35‐mediated NO production.

To further corroborate ERK kinase activity in this regulatory cascade, we conducted additional rescue experiments with an ERK‐specific inhibitor (0.5 μM) [39]. Consistent with the Raf inhibition findings, ERK blockade significantly reversed IL‐35‐mediated increases in p‐ERK, p‐eNOS (Ser1177), and NO production (Figure S3).

Collectively, these results demonstrate that IL‐35 enhances eNOS Ser1177 phosphorylation and NO production by activating the MAPK/ERK signaling pathway, and this effect is abrogated by Raf or ERK inhibition.

### 3.5 IL‐35 Activates the STAT3‐MAPK/ERK‐eNOS Signaling Axis to Promote eNOS Phosphorylation and NO Production in Endothelial Cells

Prior studies have shown that IL‐35 promotes phosphorylation of STAT1, STAT3 and STAT4, facilitating their dimerization, nuclear translocation and promoter binding to regulate downstream gene transcription. Given that STAT3 can activate the MAPK/ERK signaling pathway [40, 41], and the STRING network predicts a regulatory interaction between STAT3 and the MAPK/ERK cascade, we hypothesized that IL‐35 triggers MAPK/ERK pathway activation via STAT3. To test this hypothesis, we generated and validated si‐STAT3 constructs for subsequent experiments (Figure S4A–C).

To verify the involvement of STAT3 in IL‐35 signaling, we performed rescue experiments with si‐STAT3 in five groups: NC, PA, PA + IL‐35, PA + IL‐35+si‐NC, and PA + IL‐35+si‐STAT3. MAPK/ERK signaling activity, levels of eNOS and p‐eNOS (Ser1177), and NO production were assessed in each group. Western blotting showed that PA treatment significantly reduced phosphorylation of STAT3, eNOS, cRAF, MEK and ERK compared with controls. These reductions were reversed by IL‐35 treatment (Figure 5A–I). STAT3 silencing significantly decreased levels of p‐STAT3, total STAT3, p‐eNOS (Ser1177) and total eNOS, as well as phosphorylation of cRAF, MEK and ERK (Figure 5A–I). NO fluorescence assays further confirmed that si‐STAT3 attenuated IL‐35‐induced NO release in endothelial cells (Figure 5J,K), corroborating the pivotal role of STAT3 in IL‐35‐mediated NO production.

**Figure 5:**
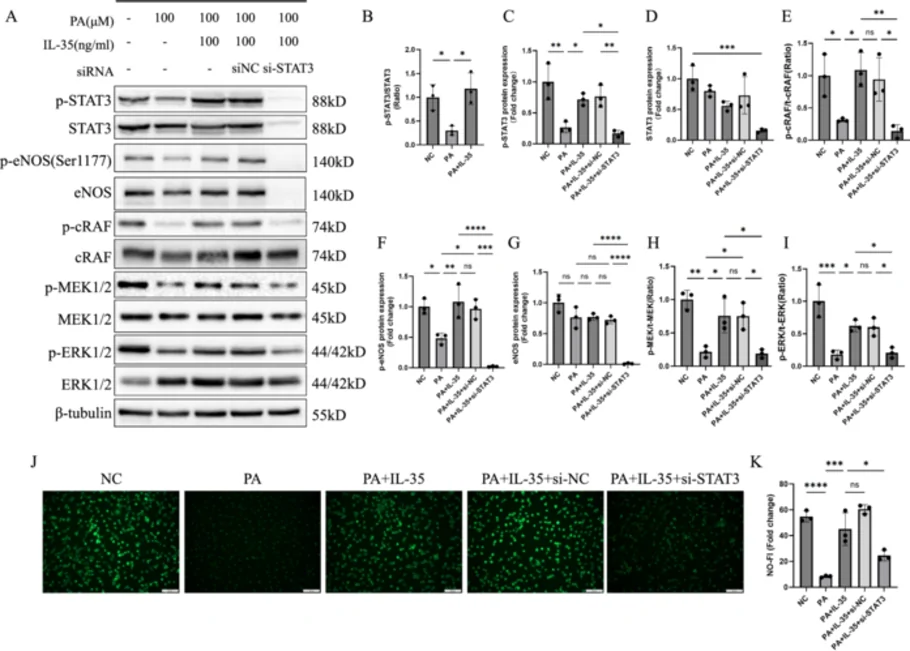
IL‐35 activates the STAT3‐MAPK/ERK‐eNOS signaling axis to promote eNOS phosphorylation and NO production in endothelial cells. (A) Western blot bands of p‐STAT3, p‐eNOS (Ser1177), p‐cRAF, p‐MEK, p‐ERK, and corresponding total proteins in HUVECs. (B–I) Densitometric analysis of p‐STAT3, STAT3, p‐eNOS (Ser1177), eNOS, p‐cRAF, p‐MEK, and p‐ERK normalized to total proteins or β‐tubulin, *n* = 3. (J–K) NO fluorescence intensity in HUVECs (J, scale bar = 50 μM) with semi‐quantification (K, *n* = 3). Data are mean ± SD. ns, not significant; *p < 0.05, **p < 0.01, ***p < 0.001, ****p < 0.0001.

Collectively, these results indicate that STAT3 suppression attenuates IL‐35‐induced MAPK/ERK pathway activation and reduces eNOS expression. IL‐35 enhances endothelial function by activating the STAT3‐MAPK/ERK‐eNOS axis to promote eNOS phosphorylation and NO synthesis.

## 4 Discussion

Obesity‐induced endothelial dysfunction, characterized by disrupted eNOS‐NO signaling and impaired endothelium‐dependent vasodilation, is a key driver of cardiometabolic disease progression. Current metabolic therapies provide vascular protection largely through indirect, metabolism‐dependent mechanisms; residual chronic inflammation and persistent endothelial dysfunction frequently persist despite optimal metabolic control, highlighting an unmet clinical need for interventions directly targeting the vascular inflammatory microenvironment and endothelial function. As an anti‐inflammatory IL‐12 family cytokine, IL‐35 exerts endothelial‐protective effects in diverse contexts, including sepsis and chronic atherosclerosis, primarily by inhibiting endothelial activation and inflammatory cell adhesion. While systemic and tissue IL‐35 levels are consistently downregulated in obesity, whether IL‐35 can mitigate obesity‐associated endothelial vasodilatory dysfunction and the underlying intracellular signaling cascades remains unclear. In this study, we demonstrate that IL‐35 ameliorates obesity‐related endothelial vasodilator dysfunction by promoting eNOS Ser1177 phosphorylation and NO production, and identify the STAT3‐MAPK/ERK‐eNOS axis as the core mediating pathway. These findings extend current understanding of IL‐35 vascular biology and support IL‐35 as a promising therapeutic candidate for metabolic vascular injury.

Notably, multiple lines of evidence support the translational relevance of our HUVEC data to the murine aortic findings. First, core endothelial regulatory pathways (eNOS activation, MAPK/ERK cascade, and STAT3 signaling) are highly conserved across both venous and arterial endothelial cells and between human and murine species [30]. Second, PA‐induced lipotoxic injury in HUVECs recapitulates key features of high‐fat diet‐induced aortic endothelial dysfunction, including reduced eNOS Ser1177 phosphorylation and decreased NO production [24]. Third, this cellular model facilitates in‐depth mechanistic analyses (e.g., IP‐MS interactomics and siRNA‐mediated silencing) that are not readily achievable in intact vascular tissue. The consistent findings across our in vitro and in vivo experiments further corroborate this translational validity.

IL‐35, an anti‐inflammatory IL‐12 family cytokine predominantly secreted by Tregs, plays a critical immunosuppressive role in conditions such as ulcerative colitis and systemic lupus erythematosus [42, 43]. In endothelial cells, it enhances endothelial function by attenuating inflammation and modulating cell adhesion and angiogenesis [17, 18, 19, 20]. Serum IL‐35 levels are markedly reduced in obesity, likely because of impaired Treg function resulting from chronic adipose tissue inflammation [16, 44]. While IL‐35 does not alter cholesterol metabolism, it significantly lowers triglyceride levels, potentially by downregulating PPARγ and C/EBPα to inhibit adipogenic differentiation and triglyceride accumulation [45].

Our study further demonstrates that serum IL‐35 is systemically reduced in obese mice, while EBI3 expression is specifically downregulated in aortic endothelial cells. Exogenous IL‐35 selectively upregulates EBI3 without affecting IL‐12A levels, similar with our previous observations in LPS‐ and hypoxia‐induced endothelial injury models [21, 46]. This divergent expression pattern is not unique to obese vascular endothelium; differential regulation of the two IL‐35 subunits is commonly observed in diverse pathological states. For example, in a murine hindlimb ischemia model, EBI3 was markedly elevated in ischemic muscle tissue whereas IL‐12A was simutaneously reduced, illustrating different regulation of the two subunits under injury conditions [6]. This non‐parallel pattern suggests EBI3 and IL‐12A may each have functions beyond their joint role as components of the IL‐35 heterodimer. Notably, free EBI3 has been identified as an independent immunosuppressive cytokine that maintains peripheral immune tolerance via the gp130/IL‐27Rα receptor axis and downstream STAT3 activation [47]. By contrast, no robust evidence supports the notion that free IL‐12A acts as a standalone ligand to activate membrane receptor signaling and elicit measurable cellular responses. Whether altered abundance of one single subunit directly modulates heterodimer assembly remains incompletely defined, and direct signaling from putative EBI3‐EBI3 homodimers lacks solid experimental support. Since the present study does not include EBI3 or IL‐12A genetic deletion experiments, we cannot formally establish how subunit imbalance shapes IL‐35 biology in obese endothelial injury, which warrants further investigation.

While Tregs are the primary source of tissue‐derived IL‐35 in diseased vascular microenvironments, endothelial cells are postulated to produce IL‐35 at lower levels [6]. Consistent with our prior work demonstrating that exogenous IL‐35 upregulates aortic and endothelial EBI3 and IL‐12A [20, 21], We speculate that endothelial cells have the potential to assemble and secrete IL‑35 heterodimers upon stimulus‑driven subunit upregulation. Nevertheless, direct proof of endothelial‐derived IL‐35 secretion remains a technical challenge. We detected no appreciable differences in secreted IL‐35 or EBI3‐IL‐12A complexes in endothelial cells using ELISA or co‐immunoprecipitation (unpublished observations). This is likely attributable to low endogenous IL‐35 production (if present), the cytokine's biochemical instability and degradation, and residual exogenous recombinant IL‐35 in culture supernatants or plasma that precludes reliable discrimination of endogenously secreted protein. Accordingly, our inference of endothelial IL‐35 biosynthesis is indirect, grounded primarily on detection of its subunits by immunofluorescence and Western blotting, rather than on direct measurement of assembled heterodimeric IL‐35.

Our results showed that ACh‐induced endothelium‐dependent vasodilation was significantly impaired in the HFD group, and IL‐35 treatment markedly ameliorated this impairment. By contrast, IL‐35 elicited only a non‐significant trend toward improvement in SNP‐induced endothelium‐independent vasodilation. These findings indicate that systemically administered exogenous IL‐35 exerts predominant protective effects on the endothelial layer, rather than a robust direct influence on medial VSMCs. This differential effect is largely attributed to anatomical barriers: the endothelial monolayer forms a functional diffusion barrier between the bloodstream and vascular media. The cytokine microenvironment of medial VSMCs is predominantly maintained by paracrine secretion from perivascular adipose tissue (PVAT) and the surrounding adventitia, rather than circulating cytokines. In our study, intraperitoneally injected IL‐35 primarily elevated circulating IL‐35 concentrations. This circulating IL‐35 preferentially acted on endothelial cells directly exposed to the bloodstream but had limited ability to penetrate the endothelial barrier to reach underlying VSMCs. Notably, obesity‐induced chronic low‐grade inflammation drives functional exhaustion of adipose tissue Tregs via excessive p38 pathway activation, which is a key driver of IL‐35 deficiency in both the systemic circulation and the PVAT microenvironment [16]. Endogenous PVAT‐derived IL‐35 is hypothesized to act on VSMCs via paracrine signaling; however, direct validation of this local effect would require tissue‐specific genetic ablation of IL‐35 in adipose tissue or Tregs, which was beyond the scope of this study. Prolonging IL‐35 exposure or developing delivery strategies targeting the vascular media might augment its regulatory effects on VSMCs function.

To delineate the upstream mechanisms of eNOS Ser1177 phosphorylation, we coupled IP‐MS with protein interaction network analysis. This approach identified ERK1 as a key differentially regulated eNOS‐binding partner. Rescue experiments with Raf and ERK inhibitors further confirmed that IL‐35 enhances eNOS Ser1177 phosphorylation and NO production by activating the MAPK/ERK pathway. Notably, IL‐35 regulates MAPK signaling in a context‐dependent manner. It suppresses excessive MAPK activation for anti‐inflammatory protection in inflammation‐dominant models such as LPS‐induced sepsis [19]. By contrast, under obesity‐associated chronic lipotoxic stress in which the Raf‐MEK‐ERK cascade is chronically repressed, IL‐35 restores this pathway to drive eNOS‐dependent NO synthesis. These bidirectional responses, shaped by pathological stimuli and baseline MAPK activity, highlight the functional plasticity of IL‐35 in endothelial homeostasis.

Furthermore, IL‐35 interacts with distinct receptor complexes and activates specific STAT transcription factors in a cell‐type‐specific manner, with each subunit binding to a separate receptor chain within the complex [48, 49]. We previously demonstrated that IL‐35 activates STAT1 and STAT4 in endothelial cells in the context of endotoxemia [21]. Here, our identification of STAT3 as the upstream activator of the MAPK/ERK‐eNOS axis suggests that obesity‐associated lipotoxic stress remodels the endothelial receptor repertoire and STAT coupling profile, driving preferential STAT3 activation. This mode of regulation constitutes a non‐canonical signaling pathway. The classical paradigm positions MAPKs as upstream kinases that phosphorylate and activate STAT proteins [50], yet accumulating evidence from other cellular contexts supports the existence of a reverse regulatory relationship [40, 41]. Collectively, these observations indicate that STAT3 and MAPK/ERK form a mutually regulated feedback loop rather than a simple unidirectional cascade, highlighting the context‐dependent functional plasticity of IL‐35 in endothelial homeostasis.

In addition, we observed that STAT3 silencing decreased total eNOS protein abundance. As a canonical transcription factor, activated STAT3 translocates to the nucleus and binds to STAT3‐binding motifs in target gene promoters. Since functional STAT3 binding sites have been identified in the eNOS promoter [51], we postulate that STAT3 sustains basal eNOS expression at the transcriptional level. This dual regulation of eNOS, which controls both total protein abundance and activating phosphorylation through the MAPK/ERK pathway, may underpin the robust protective effect of IL‐35 on the endothelial eNOS‐NO axis. Nevertheless, the direct transcriptional control of eNOS by STAT3 in this context requires further validation via chromatin immunoprecipitation assays.

This study has several limitations. First, most mechanistic experiments were performed in vitro. While our in vivo findings are consistent with our in vitro findings, it remains unclear whether these regulatory mechanisms are fully recapitulated in the complex in vivo pathological microenvironment. Second, all in vivo experiments were conducted exclusively in male mice. Sex differences are known to affect obesity‐related vascular injury and endothelial function regulation, so the generalizability of our findings across sexes requires validation in female models. Third, mouse and human vascular physiology and immune regulatory mechanisms differ fundamentally; extrapolation to human pathophysiology requires further validation in primary human arterial endothelial cells or clinical samples. Fourth, our study used only exogenous IL‐35 intervention and therefore lacked loss‐of‐function validation via gene knockout or neutralizing antibodies. Fifth, the small sample size in some experiments may have led to an overestimation of effect sizes. Finally, the high cost and instability of recombinant IL‐35 limit its clinical translation. Future studies should identify small‐molecule compounds that induce endogenous IL‐35 expression to develop more viable therapeutic strategies.

## 5 Conclusion

In summary, this study demonstrates that IL‐35 ameliorates obesity‐associated impairment of endothelium‐dependent vasodilation by activating the STAT3‐MAPK/ERK‐eNOS signaling axis, thereby enhancing eNOS Ser1177 phosphorylation and NO production. These findings establish IL‐35 as a promising therapeutic target for obesity‐related cardiovascular and metabolic complications.

## Author Contributions

**Kai Li:** conceptualization, methodology, investigation, validation, formal analysis, writing – original draft, writing – review and editing. **Jie Feng:** methodology. **Ren Gong:** formal analysis, resources. **Yunman Liu:** software. **Yanqing Wu:** conceptualization, resources, writing – review and editing, supervision, funding acquisition.

## Ethics Statement

All animal experiments were approved by the Nanchang University Laboratory Animal Ethics Committee (NCULAE‐20250901009).

## Conflicts of Interest

The authors declare no conflicts of interest.

## Supporting information


Supporting File 1

Supporting File 2

Supporting File 3

Supporting File 4

Supporting File 5

Supporting File 6

## Data Availability

The data that support the findings of this study are available from the corresponding author upon reasonable request.
