# Supplementary material for: Interleukin‐35 Ameliorates Obesity‐Associated Impairment of Endothelium‐Dependent Vasodilation via the STAT3‐MAPK/ERK‐eNOS Signaling Axis
Source: Immun Inflamm Dis. 2026 Oct 22;14(10):e70564. doi: 10.1002/iid3.70564 (PMC13689533; doi:10.1002/iid3.70564)
Supplement: Supplementary file 1 [file IID3-14-e70564-s004.docx]

PSS and KPSS solution

Preparation of 1 L PSS solution

First, prepare a 1mmol/L CaCl_2_ solution using double-distilled water and sterilize it by filtration through a 0.22 μm filter. Add 7.598 g NaCl, 0.175 g KCl, 0.08 g KH_2_PO4, 0.145 g MgSO4·7H_2_O, 1.25 g NaHCO_3_ and 1.00 g glucose. Stir continuously during preparation. Add 1.6 mL of 1mmol/L CaCl_2_, then add 200 mL of double-distilled water. Continue stirring the solution until the EDTA is completely dissolved, which takes approximately 15 minutes at room temperature. Store in a 4℃ refrigerator for later use.

Preparation of 1 L of KPSS solution

Add 4.37 g NaCl, 4.47 g KCl, 0.16 g KH_2_PO4, 0.29 g MgSO4·7H_2_O, 1.00 g NaHCO_3_, 1.00 g glucose, and 0.01 g EDTA. Stir continuously during preparation. Add 1.6 mL of 1.0 M CaCl_2_, then add 200 mL of double-distilled water. Continue stirring the solution until the EDTA is completely dissolved, which takes approximately 15 minutes at room temperature. Store at 4℃ refrigerator for later use.

si-NC (Negative Control)

Sense: 5'-UUCUCCGAACGUGUCACGUtt-3'

Antisense: 5'-ACGUGACACGUUCGGAGAAtt-3'

si-STAT3-1

Sense: 5'-GGCGUCCAGUUCACUACUA-3'

Antisense: 5'-UAGUAGUGAACUGGACGCC-3'

si-STAT3-2

Sense: 5'-AGACCCGUCAACAAAUUAA-3'

Antisense: 5'-UUAAUUUGUUGACGGGUCU-3'

si-STAT3-3

Sense: 5'-CAUCGAGCAGCUGACUACA-3'

Antisense: 5'-UGUAGUCAGCUCGCGAUG-3'

Primer

GAPDH-F: GCACCGTCAAGGCTGAGAAC

GAPDH-R: TGGTGAAGACGCCAGTGGA

STAT3-F: ACAGGTTGGACATGATGCACAC

STAT3-R: TAAGCCTTTGCCCTGCATGA
