# Supplementary material for: Interleukin‐35 Ameliorates Obesity‐Associated Impairment of Endothelium‐Dependent Vasodilation via the STAT3‐MAPK/ERK‐eNOS Signaling Axis
Source: Immun Inflamm Dis. 2026 Oct 22;14(10):e70564. doi: 10.1002/iid3.70564 (PMC13689533; doi:10.1002/iid3.70564)
Supplement: Supplementary file 2 [file IID3-14-e70564-s006.docx]

Raw Ct values of GAPDH for reference gene stability validation

| Group | GAPDH Ct replicates | Mean Ct | SD | CV (%) |
| --- | --- | --- | --- | --- |
| NC | 14.29, 14.28, 14.54 | 14.37 | 0.15 | 1.02 |
| si-NC | 14.33, 14.25, 14.12 | 14.23 | 0.11 | 0.74 |
| si-STAT3-1 | 15.05, 15.05, 14.93 | 15.01 | 0.07 | 0.46 |
| si-STAT3-2 | 14.47, 14.41, 14.27 | 14.38 | 0.10 | 0.71 |
| si-STAT3-3 | 14.73, 14.73, 14.71 | 14.72 | 0.01 | 0.08 |
| Overall | — | 14.54 | 0.31 | 2.10 |
